# The Relationship between Virtual Self Similarity and Social Anxiety

**DOI:** 10.3389/fnhum.2014.00944

**Published:** 2014-11-19

**Authors:** Laura Aymerich-Franch, René F. Kizilcec, Jeremy N. Bailenson

**Affiliations:** ^1^Virtual Human Interaction Lab, Department of Communication, Stanford University, Stanford, CA, USA

**Keywords:** virtual reality, virtual environment, social anxiety, public speaking, virtual self, self-representation, self-image, virtual classroom

## Abstract

In virtual reality (VR), it is possible to embody avatars that are dissimilar to the physical self. We examined whether embodying a dissimilar self in VR would decrease anxiety in a public speaking situation. We report the results of an observational pilot study and two laboratory experiments. In the pilot study (*N* = 252), participants chose an avatar to use in a public speaking task. Trait public speaking anxiety correlated with avatar preference, such that anxious individuals preferred dissimilar self-representations. In Study 1 (*N* = 82), differences in anxiety during a speech in front of a virtual audience were compared among participants embodying an assigned avatar whose face was identical to their real self, an assigned avatar whose face was other than their real face, or embodied an avatar of their choice. Anxiety differences were not significant, but there was a trend for lower anxiety with the assigned dissimilar avatar compared to the avatar looking like the real self. Study 2 (*N* = 105) was designed to explicate that trend, and further investigated anxiety differences with an assigned self or dissimilar avatar. The assigned dissimilar avatar reduced anxiety relative to the assigned self avatar for one measure of anxiety. We discuss implications for theories of self-representation as well as for applied uses of VR to treat social anxiety.

## Introduction

Virtual reality (VR) enables people to experience an alternate reality. Transforming the appearance of one’s self is a particularly powerful application of VR. This can be achieved by modifying the appearance of one’s avatar (Biocca, 1997; Bailenson and Blascovich, 2004), thereby providing the person with a new self-representation. People are known to psychologically identify with virtual representations that do not necessarily reflect their actual appearances (Kim, 2011). Thus, in virtual worlds, people can explore different versions of their self and become someone else (Turkle, 1995). Moreover, the appearance of avatars can cause behavioral and attitudinal shifts (Yee and Bailenson, 2006, 2007, 2009; Vasalou et al., 2007; Groom et al., 2009; Ahn and Bailenson, 2011; Hershfield et al., 2011; Peck et al., 2013). Also, observing the behavior of doppelgangers – virtual representations that resemble the self in appearance but behave independently – can influence attitudes and behavior (Fox and Bailenson, 2009, 2010; Fox et al., 2012; Aymerich-Franch and Bailenson, 2014).

In light of its psychological effects, VR is uniquely positioned to support the treatment of phobias and other anxiety disorders (Wiederhold and Wiederhold, 2000). In particular, the possibility to transform self-appearance offers a unique opportunity to treat social anxiety, one of the most common anxiety–mood disorders worldwide (Kessler et al., 2012). Social anxiety is an intense fear of negative evaluation from others in social or performance situations (4th ed., text rev.; DSM–IV–TR; American Psychiatric Association, 2000). According to cognitive models of social phobia, negative self-images play an important role in maintaining social anxiety (Izgiç et al., 2004; Stopa and Jenkins, 2007) as socially anxious individuals create a negatively distorted mental representation of their ostensible appearance toward others. People who suffer from social anxiety selectively attend to and magnify negative aspects of their ostensible public image, which may also be influenced by past failures in social situations (Clark and Wells, 1995). However, previous studies that have used VR to address social anxiety have focused on manipulating features of the audience (Pertaub et al., 2001, 2002; James et al., 2003; Garau et al., 2005; Slater et al., 2006; Rinck et al., 2010; Wieser et al., 2010; Cornwell et al., 2011; Pan et al., 2012), but have not transformed the self in a social situation. Similarly, studies that have used virtual reality exposure therapy (VRET) to treat social phobia generally recreate social virtual environments for exposure (Harris et al., 2002; Roy et al., 2003; Anderson et al., 2005; Klinger et al., 2005; Wallach et al., 2011), without attempting to modify patients’ self-representations.

Current VR therapies for social anxiety could incorporate transformations of the virtual self in order to restructure patients’ distorted self-image in combination with exposure. We believe that having a virtual self-representation dissimilar to the real self in a social situation might decrease anxiety, because (a) virtual embodiment through an avatar can significantly alter a person’s body schema and social role (Biocca, 1997; Kilteni et al., 2012), and (b) a dissimilar virtual self provides anonymity, which reduces inhibition and anxiety and facilitates self-expression. Embodying a dissimilar self could thereby neutralize some of the factors that contribute to social anxiety.

In this paper, we examine the effect of an altered self-representation in VR on social anxiety and present a new approach to treat social anxiety using VR. In order to explore the effect of the appearance of the virtual self on social anxiety, we conducted three studies. In a pilot study, we examined the relationship between avatar similarity preference and trait public speaking anxiety during an imagined virtual speech task. We expected that higher social anxiety would be associated with a stronger preference for a dissimilar avatar. Based on the results of the pilot study, we designed an experiment (Study 1) in which we used immersive VR to manipulate appearance similarity of participants’ virtual self-representations to their physical appearances in a public speaking context. Since multisensory correlations are essential to experience embodiment (Botvinick and Cohen, 1998), we added a virtual mirror in the virtual environment and synchronized participant’s body movement to the avatar reflection in the mirror in order to create embodiment and identification with the virtual body. Prior work using VR suggests that real-time virtual mirror reflections of upper body movements contribute to feelings of body ownership (González-Franco et al., 2010). We expected that participants with an avatar matching their real appearance would experience higher anxiety compared to participants with a dissimilar self-representation. In Study 1, we also examined the effect on anxiety of choosing the appearance of the virtual self in comparison to being assigned to an avatar in a public speaking situation. Prior work has examined the effects of avatar choice in online environments. The findings suggest that choosing the appearance of the virtual self in virtual environments affects constructs related to anxiety and self-consciousness. In particular, previous work has found increased self-awareness during online interactions with other people for users who were represented by an avatar that matched their appearance and preferences compared to users without an avatar representation (Vasalou et al., 2007). In addition, giving players the possibility of choosing the character that represents them during an online game was found to induce greater arousal compared to not having the option to choose (Lim and Reeves, 2009). In the first two experimental conditions, participants were assigned the avatar. In order to examine the effect on anxiety of choosing an avatar relative to being assigned one, we added a third condition, in which participants were able to choose their self-representation. To test our hypothesis and research questions, we compared anxiety outcomes among participants embodying an assigned avatar looking like the real self, an assigned avatar looking dissimilar to the real self, or an avatar of their choice, during a speech in front of a virtual audience. In Study 2, we partially replicated Study 1, comparing participants with an assigned similar versus dissimilar appearance with a larger sample and a more established measure of anxiety. In our experimental studies, we also examined effects on the sense of presence, the psychological state in which virtual objects are experienced as actual objects in either sensory or non-sensory ways (Lee, 2004). Presence is an important factor to consider in studies that explore phobia-related issues using VR, since it contributes to the experience of anxiety in a virtual environment (Price and Anderson, 2007).

## Pilot Study

We designed a survey in which participants had to choose an avatar to embody if they were going to give a speech in VR. They indicated how similar the avatars would be to their physical selves. We predicted that participants with higher levels of trait social anxiety would prefer to embody dissimilar avatars compared to participants with lower levels of trait social anxiety:

H_1_. Avatar similarity and social anxiety correlate negatively, i.e., higher social anxiety is associated with a stronger preference for a dissimilar avatar.

### Method

#### Participants

A total of 252 participants from the United States completed the survey. The sample was composed of 64% males, aged between 18 and 74 years, with 49% of the sample between the ages of 25 and 34 years. Participants were recruited through Amazon’s Mechanical Turk crowdsourcing service and received a $1 payment for completing the survey. Mechanical Turk has been widely used in previous studies to recruit participants and has been shown to provide data comparable to more traditional methods of recruitment (Kittur et al., 2008; Golbeck and Fleischmann, 2010; Sprouse, 2011; Liu et al., 2012; Aker et al., 2013).

#### Design

In the survey, participants first answered questions regarding socio-demographic variables and interactive media habits (video-gaming, virtual worlds, VR). Public speaking anxiety was measured using the *Personal Report of Communication Apprehension* (PRCA-24; McCroskey, 1982), a 24-item scale. Participants rated their answers on a five-point scale ranging from *strongly disagree* (1) to *strongly agree* (5). The reliability of this measure was α = 0.95 and the average score on this test was *M* = 69.56 (SD = 22.27).

Next, participants reviewed a passage describing VR, an avatar, technologies such as head-mounted displays (HMD), the content of a virtual scene, and the concept of similarity in self-representation. The passage included both verbal description and images.

Participants were informed that they would be giving a speech on two different topics that may or may not be socially sensitive in nature. For the first topic, participants would discuss their favorite vacation (i.e., “imagine that you are in this virtual classroom full of people. You are required to deliver a speech about your favorite vacation in front of the virtual audience”). For the other topic, they would deliver a speech on a sensitive social issue (i.e., “imagine that you are in this virtual classroom full of people. You are required to give a speech about a sensitive social issue in front of the virtual audience”).

Participants rated each situation with an avatar similarity question (i.e., “if you had to design your own avatar for this task, how similar to your real appearance would you make your avatar?”). The question was rated on a five-point scale ranging from *extremely similar* (1) to *not at all similar* (5). We included a picture of a virtual classroom to accompany these questions. Across the two situations, the two ratings correlated highly (*r* = 0.78, *t*_252_ = 19.8, *p* < 0.001), so we created an index of avatar similarity by averaging across the two situations.

### Results and Discussion

There was a significant negative correlation between avatar similarity and social anxiety. Avatar similarity correlated significantly with the PRCA-24 across the two situations (*r* = −0.43, *t*_252_ = 7.6, *p* < 0.001) and for each situation individually: favorite vacation (*r* = −0.37, *t*_252_ = 6.4, *p* < 0.001) and sensitive social issue (*r* = 0.44, *t*_252_ = 7.8, *p* < 0.001). As predicted, the higher social anxiety, the stronger the preference for embodying a dissimilar rather than a similar avatar during a speech in VR.

## Study 1

Since the results of our pilot study suggested that a dissimilar virtual self in a public speaking task in VR is associated with lower anxiety, we designed an experiment to examine whether becoming someone else would reduce anxiety in a public speaking situation. In order to examine the consequences of avatar choice on public speaking anxiety in comparison to an assigned avatar, we added a third condition to the experimental design in which participants chose the appearance of their virtual self. We hypothesized that participants embodying a self avatar during a public speaking task in VR would experience greater levels of anxiety than participants with a dissimilar avatar. In particular, we made the following prediction:

H_1_. During a speech in front of a virtual audience, participants with an avatar of their own face (*self* condition) will experience higher levels of self-perceived physiological sensations and state anxiety than participants with an avatar of a face dissimilar to their own (*other* condition).

We also formulated the following research question regarding the possibility of choosing the avatar:

RQ_1_. Do participants in the *choice* condition experience different levels of anxiety than those in the *self* and *other* conditions?

Regarding presence, we formulated the following research question:

RQ_2_. Do participants in the *self*, *other*, and *choice* condition experience different levels of self, social, and spatial presence?

### Method

#### Participants

Eighty-eight participants attending an American university took part in the experiment. We discarded six due to technical failure or motion sickness. The final sample consisted of 82 experimental subjects (51 females and 31 males) aged 18–32 years (*M* = 20.18, SD = 1.88).

#### Design

**Figure 1:**
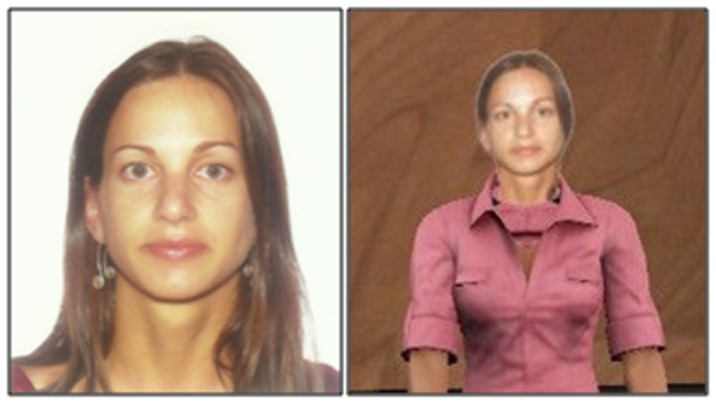
**A participant and her avatar modeled from a photograph of her, in the self face condition**.

Participants were assigned into one of three experimental conditions: *self*, *other*, or *choice*. In the *self* condition, participants embodied an avatar with their own face modeled after a photograph (Figure 1). In the *other* condition, participants were assigned a dissimilar face modeled after a previous participant’s photograph. It was ensured that the avatar face in the *other* condition matched participants’ sex and skin color by pairing each participant in the *other* condition with a previous participant from the same study of the same sex and with similar skin color. The avatar faces did not vary across conditions, as faces from the *self* condition were reused in the *other* condition. Faces in the *other* condition were paired with ones from the *self* condition in one of eight categories defined by sex (male/female) and skin color (lightest to darkest). This ensured that faces had comparable objective features in the *self* and *other* conditions. In the *choice* condition, we showed participants a chart that contained 18 photographs of people of their same sex and asked them which avatar would choose to represent them if they had to give a speech in front of a virtual audience. Participants were assigned the face they chose.

#### Procedure

**Figure 2:**
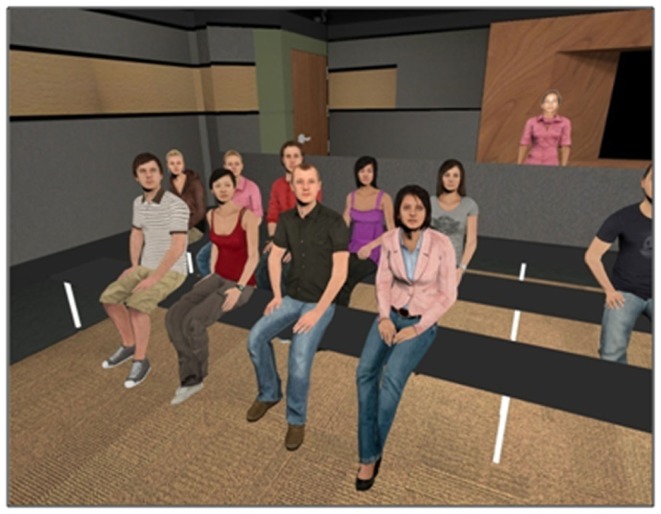
**Virtual audience with participant’s avatar reflection at the back of the classroom during the speech**.

Participants completed the experiment individually. When they arrived, we took a picture of the participants’ face. In the *self* condition, we modeled the pictures of the participants’ face to become their avatar’s head. In the *choice* condition, participants looked at a chart with faces and had to choose which person they would like to become their avatar if they had to give a speech in front of an audience. Then, in all conditions, they filled out a pre-survey. After that, we required them to improvise a 3-min speech in front of a virtual audience. They were able to decide the topic. As a possibility, we suggested them to talk about a hobby or interest. Once in the experimental room, participants wore an HMD and tracking sensors on their head and wrists. In the virtual world, participants saw a curtain that opened and an empty classroom appeared. We told subjects that an avatar would represent them in the virtual environment and asked them to look at a virtual mirror placed on the back wall of the room. We told them to lift their arms one at a time to make sure that they were aware of their avatar’s self-representation, which moved its hands accordingly in real time. Participants were also asked to describe their avatar briefly. Then, the curtain closed and they were told that the audience would arrive at the classroom shortly. We asked them to rate their anxiety before the speech. After a few seconds, the curtain opened again to reveal the seated virtual audience, watching the participant. The audience was composed of 12 agents (6 males and 6 females) of various races as depicted in Figure 2. The agents in the audience kept neutral faces during the speech. They looked at the participant most of the time. Also, we programed them to perform some stock idling gestures such as slightly moving their heads or arms from time to time for a realistic appearance. Once the curtain was fully open, participants started their speech. Participants were able to see their virtual representation at all times during the performance, which mirrored their head, arm, and body movements. After they concluded, the curtain closed. We asked them to rate how anxious they felt during the speech. Then, we helped them to take off the HMD. Finally, they completed a post-survey and were thanked for their participation.

#### Apparatus

**Figure 3:**
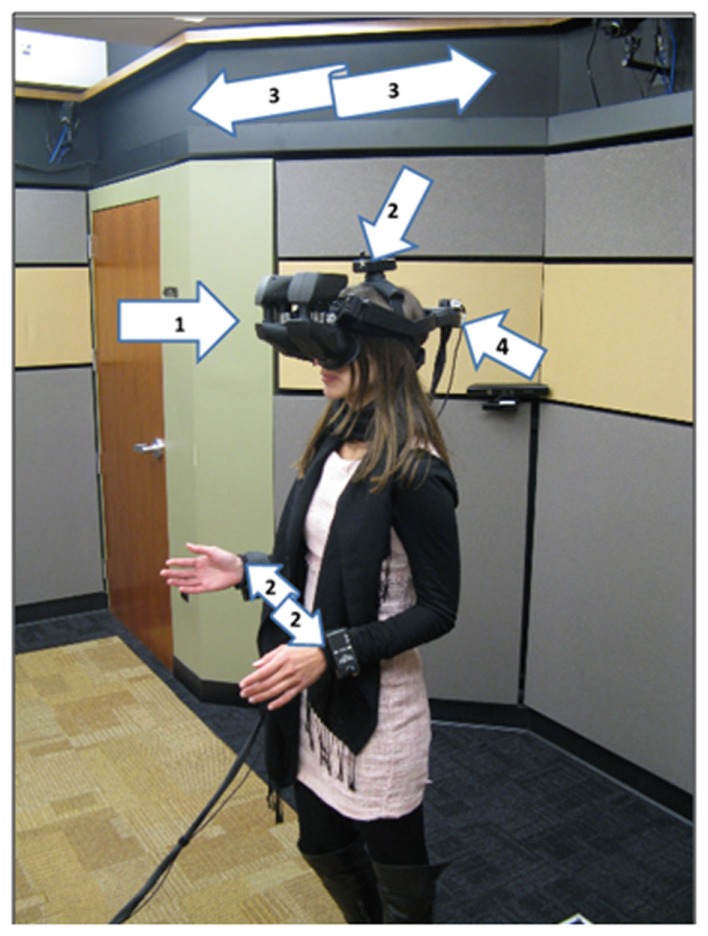
**Participant wearing the HMD (1), tracking sensors on the head and wrists (2), cameras (3) to detect the position of the trackers, and orientation device (4), during the speaking task**.

We created the virtual classroom using Worldviz’s Vizard VR Toolkit. Participants wore an nVisor SX111 head-mounted display (NVIS, Reston, VA, USA) with a resolution of 2560 horizontal and 1024 vertical and a refresh rate of 60 frames per second to visualize the virtual world. An optical tracking system (Worldviz PPT-E) combined with an orientation sensor (Intersense3 Inertial Cube) provided total tracking of six degrees of freedom (x, y, z position and pitch, yaw, and roll) for the head. The participants wore trackers on the hands that tracked the x, y, z position of each hand (but not orientation) as well (Figure 3).

### Measures

#### Pre-screen

We only invited participants who scored six or higher on the Mini-SPIN test (Connor et al., 2001), a screening test for social anxiety consisting of three questions.

#### Pre-test survey measures

Trait public speaking anxiety was assessed using the Personal Report of Communication Apprehension (PRCA-24; McCroskey, 1982). The reliability of this test in the study was α = 0.90 and the average score was *M* = 71.73 (SD = 14.39). PRCA-24 scores were not significantly different across conditions: *M*_self_ = 70.93, *M*_other_ = 71.31, *M*_choice_ = 72.93 (SD_self_ = 12.63, SD_other_ = 15.75, SD_choice_ = 15.11); *F*_2, 79_ = 0.15, *p* = 0.86 based on an ANOVA.

In addition, trait social anxiety was measured using the *Brief Fear of Negative Evaluation Scale* (B-FNE; Leary, 1983). This scale is often used to assess fear of negative evaluation, the core feature of social anxiety disorder (Weeks et al., 2005). This measure yielded a reliability of α = 0.86 and the average score was *M* = 3.26 (SD = 0.64). B-FNE scores were not significantly different across conditions: *M*_self_ = 3.33, *M*_other_ = 3.15, *M*_choice_ = 3.29 (SD_self_ = 0.64, SD_other_ = 0.65, SD_choice_ = 0.65); *X*2*_df_*_ = 2_ = 1.5, *p* = 0.48 based on Kruskal–Wallis test (residual errors not normally distributed).

#### Post-test survey measures

Participants rated anxiety before and during the speech for how anxious they felt before and during the speech, using a 0 (no anxiety) to 100 (extreme anxiety) scale (Stopa and Jenkins, 2007). Participants answered these questions while they were in the virtual world. The average score on this measure was *M* = 42.68 (SD = 24.97) for anxiety before the speech and *M* = 46.21 (SD = 25.71) for anxiety during the speech.

The *Body Sensations Questionnaire* (BSQ; Chambless et al., 1984) was used to measure self-perceived physiological sensations. This measure is a 17-item scale that comprises items concerning sensations associated with autonomic arousal. Participants rate how intensely they experienced each sensation (e.g., heart palpitations or dry throat) during the speech on a five-point scale, ranging from *not at all* (1) to *extremely* (5). The BSQ has been previously used in public speaking anxiety studies (McCullough et al., 2006). The BSQ yielded a reliability of α = 0.88 and the average score was *M* = 1.73 (SD = 0.58).

A 15-item presence scale consisting of five items for self-presence (e.g., to what extent did you feel that the avatar’s body was your own body?), five items for social presence (e.g., to what extent did you feel that the audience was present?), and five items for spatial presence (e.g., to what extent did you feel that the virtual classroom seemed like the real world?) was adapted from presence scales used in previous studies (Nowak and Biocca, 2003; Bailenson and Yee, 2007; Fox et al., 2009). The items were rated on a five-point scale ranging from *very highly* (1) to *not at all* (5). For self-presence, social presence, and spatial presence, the reliability was α = 0.91, α = 0.93, and α = 0.87, respectively. Overall, presence was computed by averaging over the three presence dimensions. The reliability of the overall presence measure was α = 0.92. Presence scores were reversed for better interpretability, such that high presence scores indicate a strong sense of presence (scores range from 1 to 5). The average score on these measures was *M* = 2.13 (SD = 0.90) for self-presence, *M* = 3.27 (SD = 0.96) for social presence, *M* = 2.81 (SD = 0.83) for spatial presence, and *M* = 2.74 (SD = 0.73) for overall presence.

Participants also rated the similarity of their avatar’s face with their own face as a manipulation check. The exact question wording was “when you looked at your avatar in the mirror, how similar was its face to yours?” A five-point scale from *extremely similar* (1) to *not at all similar* (5) was used. Similarity ratings were significantly higher in the *self* condition (*M* = 1.9, SD = 0.89) than in the *other* condition (*M* = 4.3, SD = 0.74; *t*_51_ = 11, *p* < 0.001, *d* = 3.0). Ratings in the *choice* condition were closer to the *other* than *self* condition (*M* = 3.9, SD = 0.89).

### Results

Descriptive and inferential statistics for anxiety and presence are summarized in Table 1. Differences between experimental conditions were tested using ANOVAs where the condition that residual errors are normally distributed was not significantly violated. The assumption was violated for measures of anxiety before and during the speech, BSQ, and self-presence. We tested differences using the non-parametric Kruskal–Wallis test for these measures. Trait anxiety (PRCA-24 and B-FNE) and state anxiety (BSQ, anxiety before and during the speech) were all correlated, except for PRCA-24 with BSQ. Presence measures (self, social, spatial, and overall) were also all correlated among them (see Table S1 in the Supplementry Material, for correlations between all measures).

**Table 1:** **Means (SD) and unadjusted statistical tests for each dependent variable**.

	Anxiety before speech^a^	Anxiety during speech^a^	BSQ^a^	Self-presence^a^	Social presence	Spatial presence	Overall presence
Self (*N* = 28)	45.64 (22.56)	49.75 (25.37)	31.25 (10.35)	2.14 (0.75)	3.69 (0.75)	3.05 (0.86)	2.96 (0.58)
Choice (*N* = 28)	41.75 (26.00)	45.29 (27.58)	28.93 (10.65)	1.98 (0.98)	2.94** (1.09)	2.56** (0.78)	2.50* (0.81)
Other (*N* = 26)	39.85 (26.20)	42.38 (24.29)	27.08* (7.08)	2.29 (0.98)	3.18** (0.87)	2.82 (0.82)	2.76 (0.74)
Test statistic	$X_{\mathrm{df}=2}^{2}=0.738$	$X_{\mathrm{df}=2}^{2}=1.07$	$X_{\mathrm{df}=2}^{2}=2.66$	$X_{\mathrm{df}=2}^{2}=2.38$	*F*_2, 79_ = 4.77	*F*_2, 79_ = 2.46	*F*_2, 79_ = 2.97
*p*	0.69	0.58	0.26	0.30	0.01	0.09	0.06

**Significantly different from the *self* condition at *p* < 0.05, *p < 0.10.

*^a^Residual errors were not normally distributed, hence a non-parametric test was employed instead of an ANOVA*.

We tested H_1_ and addressed RQ_1_ about differences in anxiety and BSQ with a simple test of unadjusted means (Kruskal–Wallis tests in Table 1) and a covariate-adjusted regression model (Table 2). Similar studies (Felnhofer et al., 2012; Aymerich-Franch and Bailenson, 2014) highlighted the relevance of sex and trait social anxiety (B-FNE) as moderators of the effect of virtual experiences on anxiety-related measures. Accordingly, we report results from two regressions for each outcome, one without covariates and one with sex and B-FNE in the model. The data provided some evidence for H_1_ that anxiety is lower with a dissimilar avatar than a self avatar based on BSQ scores (*p* < 0.10), but not for anxiety measured before and during the speech. B-FNE was a significant covariate in all regressions of anxiety and BSQ, though sex was not significant (Table 2). Regarding RQ_1_, anxiety measures were not significantly different in the *choice* condition, neither based on unadjusted tests (Table 1) nor covariate-adjusted regressions (Table 2). Average levels of anxiety in the *choice* condition were between those in the *other* and *self* condition based on descriptive statistics only. As PRCA-24 was highly correlated with B-FNE, only one could be included in the regression model, but results were qualitatively similar with PRCA-24 as a covariate in the model.

**Table 2:** **Linear regression coefficients (robust standard errors) for anxiety outcomes with two models**.

	Anxiety before speech		Anxiety during speech		BSQ (log_10_)	
	(1)	(2)	(3)	(4)	(5)	(6)
Intercept	45.6** (4.18)	47.29** (4.43)	49.75** (4.71)	50.93** (4.94)	0.244** (0.025)	0.240** (0.025)
Condition: choice	−3.89 (6.39)	−3.60 (5.81)	−4.46 (6.95)	−4.16 (6.46)	−0.037 (0.037)	−0.034 (0.033)
Condition: other	−5.80 (6.55)	−3.51 (5.96)	−7.37 (6.63)	−5.20 (6.24)	−0.055* (0.033)	−0.041 (0.031)
Sex: male	–	−6.53 (5.18)	–	−5.22 (5.64)	–	−0.004 (0.026)
B-FNE	–	13.46** (4.16)	–	12.67** (4.11)	–	0.079** (0.021)
Adj. *R*^2^	−0.015	0.125	−0.011	0.094	0.006	0.142
*F*	0.38	3.90	0.56	3.09	1.26	4.34
df	2, 79	4, 77	2, 79	4, 77	2, 79	4, 77
*p*	0.68	0.006	0.57	0.02	0.29	0.003

*Residual errors were normally distributed in all covariate-adjusted models. BSQ was log-transformed to fit a linear model, and B-FNE was centered for interpretability*.

**Significant coefficient with *p* < 0.01, *significant coefficient with *p* < 0.10.

We examined RQ_2_ about differences in types of perceived presence between conditions with ANOVAs and non-parametric tests depending on the distribution of the data (Table 1). Social, spatial, and overall presence were significantly lower in the *choice* condition than in the *self* condition (*t*_54_ > 2.2, *p* < 0.05, Cohen’s *d* = 0.79, 0.59, 0.66, respectively), but there were no significant differences in self-presence. Only social presence was lower in the *other* condition than the *self* condition, *t*_52_ = 2.3, *p* = 0.03, *d* = 0.62.

In sum, Study 1 showed that assigning participants a dissimilar face did not significantly reduce their anxiety during a speech in front of a virtual audience. However, differences in all three anxiety measures were marginally significant for BSQ and in the hypothesized direction, i.e., lower anxiety with a dissimilar than with the own face. Participants who chose their avatar experienced significantly lower levels of social, spatial, and overall presence than those who were assigned the own face. Yet, choosing an avatar was not found to induce significantly different levels of anxiety than being assigned a *self* or *other* avatar. We attempted to replicate the effect of assigning the own face or a dissimilar face in Study 2 with a larger sample size and a more established measure of anxiety to test if embodying a new self could reduce social anxiety.

## Study 2

In this study, we partially replicated Study 1 where exploratory results indicated that participants who were assigned a dissimilar face experienced marginally lower anxiety than participants who were assigned the own face, although the preliminary results yielded no significant difference in anxiety.

In order to improve the design of Study 1, a series of modifications were made in Study 2. First, a larger sample size was used to gain more statistical power to identify significant differences between conditions. A power calculation suggests that the sample size used in Study 2 could identify an effect size of 0.57 SD with 80% power and 95% confidence. Moreover, since reported anxiety before and during the public speaking situation was potentially not sensitive enough to detect significant differences in anxiety, we opted for a more established measure of anxiety, namely *the State Trait Anxiety Inventory (STAI)* (STAI; Spielberger et al., 1970, 1983). Also, participants were required to give a longer speech and had time to prepare it in order to ensure that the experience was long enough to provoke anxiety.

In line with Study 1, we hypothesized significant differences between the *self* and *other* conditions:

H_1_. During a speech in front of a virtual audience, participants with an avatar of their own face will experience higher levels of self-perceived physiological sensations and state anxiety than participants with an avatar of a face dissimilar to their own.

We also explored differences in the sense of presence between the three conditions. Accordingly, we formulated the following research question:

RQ_1_. Do participants in the *self* and *other* condition experience different levels of self, social, and spatial presence?

### Method

#### Participants

One hundred and fourteen participants attending an American university took part in the experiment. Nine participants were omitted from analysis due to technical failure or motion sickness. The final sample consisted of 105 experimental subjects (61 males and 44 females) aged 18–39 years (*M* = 20.41, SD = 2.43).

#### Design

Participants were either assigned an avatar with the own face or one with a dissimilar face. Participants in the *self* condition were assigned an avatar with a face that was modeled after their photograph, while those in the *other* condition were assigned an avatar with the face of a previous participant. The procedure was identical to the one described in Study 1.

#### Procedure

Participants completed the experiment individually. First, they filled out a pre-survey. Then, we read aloud a set of instructions, which required them to give a 5-min speech about their university in front of a virtual audience. We gave them 5 min to prepare the speech. Once in the experimental room, participants wore an HMD and tracking sensors on their head and wrists, which tracked orientation and translation of head position as they moved about the room and translation of their hands. In the virtual world, participants saw a curtain that opened and an empty classroom appeared. We told subjects that an avatar would represent them in the virtual environment and asked them to look at a virtual mirror placed on the back wall of the room. We told them to lift their arms one at a time to make sure that they were aware of their avatar’s self-representation, which moved its hands accordingly in real time. Then, the curtain closed and we told them that the audience would arrive at the classroom shortly. After a few seconds, the curtain opened again and the virtual audience was sitting, watching the participant. The audience was the same as from Study 1. Once the curtain was fully open, participants delivered their speech. Participants were able to see their virtual representation at all times during the performance, which mirrored their head, arm, and body movements. After they concluded, the curtain closed and we helped them to take off the HMD. Finally, they completed a post-survey and were thanked for their participation and debriefed.

#### Apparatus

We used the same apparatus described in Study 1.

#### Measures

##### Pre-test survey measures

Trait public speaking anxiety was assessed with the *Personal Report of Communication Apprehension* (PRCA-24; McCroskey, 1982) scale. Participants rated 24 items on a 5-point scale ranging from *strongly disagree* (1) to *strongly agree* (5). The reliability of this measure was α = 0.96 and the average score was *M* = 66.36 (SD = 17.54). PRCA-24 scores were not significantly different across conditions: *M*_self_ = 65.33, *M*_other_ = 67.64 (SD_self_ = 16.60, SD_other_ = 18.73); *F*_1, 103_ = 0.45, *p* = 0.51 based on an ANOVA.

In addition, trait social anxiety was measured using the Brief Fear of Negative Evaluation Scale (B-FNE; Leary, 1983). Participants rated 12 items on a 5-point scale ranging from *not at all characteristic of me* (1) to *extremely characteristic of me* (5). This measure yielded a reliability of α = 0.92 and an average score of *M* = 37.97 (SD = 10.22). B-FNE scores were not significantly different across conditions: *M*_self_ = 37.19, *M*_other_ = 38.94 (SD_self_ = 10.05, SD_other_ = 10.45); *F*_1, 103_ = 0.76, *p* = 0.39 based on an ANOVA.

##### Post-test survey measures

State anxiety was measured using the STAI – Form Y-1 (Spielberger et al., 1970, 1983). Participants rated how they felt (e.g., calm, tense) in a particular situation (i.e., during a speech) on a four-point scale ranging from *not at all* (1) to *very much so* (4). This portion of the scale was designed to assess transitory anxiety and it is the most commonly used measure of public speaking state anxiety in empirical studies published in Communication (Behnke and Sawyer, 2004). The reliability of the STAI was α = 0.93 and the average score was *M* = 42.6 (SD = 11.38).

Self-perceived physiological sensations were assessed using the *Body Sensations Questionnaire* (BSQ; Chambless et al., 1984). The BSQ yielded a reliability of α = 0.90 and the average score was *M* = 25.42 (SD = 9.16).

The same 15-item presence scale used in Study 1 was administered in this study. For self-presence, social presence, spatial presence, and overall presence, the reliability was α = 0.89, α = 0.90, α = 0.88, and α = 0.93, respectively. The average score was *M* = 2.43 (SD = 0.88) for self-presence, *M* = 3.42 (SD = *0*.87) for social presence, *M* = 3.20 (SD = *0*.86) for spatial presence, and *M* = 3.02 (SD = *0*.75) for overall presence.

Participants also rated the similarity of their avatar’s face with their own face as a manipulation check on the same scale used in Study 1. Similarity ratings were significantly higher in the *self* condition (*M* = 2.1, SD = 1.03) than in the *other* condition (*M* = 4.0, SD = 0.88; *t*_103_ = 9.7, *p* < 0.001, *d* = 1.9).

### Results

Descriptive and inferential statistics for anxiety and presence are summarized in Table 3. Differences between experimental conditions were tested using ANOVAs where the condition that residual errors are normally distributed was not significantly violated (all but BSQ and self-presence). A non-parametric Mann–Whitney test was used instead for these measures. Trait public speaking anxiety (PRCA-24) correlated significantly with all types of presence. Trait social anxiety (B-FNE) correlated with spatial presence, but did not correlate significantly with self, social, or overall presence. STAI and BSQ did not correlate with any type of presence. Trait anxiety (PRCA-24 and B-FNE) and state anxiety (STAI and BSQ) measures were all correlated with one another (see Table S2 in the Supplementry Material, for correlations between all measures).

**Table 3:** **Means (SD) and unadjusted statistical tests for each dependent variable**.

	STAI	BSQ^a^	Self-presence^a^	Social presence	Spatial presence	Overall presence
Self (*N* = 58)	43.69 (11.22)	26.64 (10.21)	2.60 (0.89)	3.41 (0.89)	3.20 (0.89)	3.07 (0.77)
Other (*N* = 47)	41.27 (11.57)	23.93 (7.51)	2.22 (0.83)	3.43 (0.86)	3.19 (0.84)	2.95 (0.72)
	*t*_103_ = 1.08, *p* = 0.283	*W* = 1617, *p* = 0.102	*W* = 1011, *p* = 0.023	*t*_103_ = 0.12, *p* = 0.906	*t*_103_ = 0.08, *p* = 0.940	*t*_103_ = 0.85, *p* = 0.395

*^a^Residual errors were not normally distributed, hence a non-parametric test was employed instead of a *t*-test*.

Hypothesis 1 that participants with the own face would experience greater anxiety than those with a dissimilar face was examined with unadjusted and covariate-adjusted comparisons of STAI and BSQ levels. Due to the highly skewed distribution of BSQ scores, we employed a non-parametric test in the unadjusted comparison and resorted to a negative binomial model for the covariate-adjusted regression, as a simpler log-linear or Poisson model did not fit the data sufficiently well. The same set of covariates as in Study 1 was included, sex and B-FNE.

Anxiety measured by BSQ was significantly reduced by 14% [95% CIs = (0.9%, 27%)] in the *other* condition based on the covariate-adjusted regression model (Table 4). Sex and B-FNE were significant covariates, with lower anxiety for males than females at the same order of magnitude as the experimental manipulation. B-FNE contributed positively to BSQ in the model. There was no significant interaction effect between the experimental assignment and sex or B-FNE (*z* < 1.0, *p* > 0.30). In contrast to B-FNE, anxiety measured by STAI was not significantly lower with a dissimilar than with the own face, neither in the unadjusted test (Table 3) nor the covariate-adjusted test (Table 4).

**Table 4:** **Regression coefficients (robust standard errors) for anxiety dependent variables with two models**.

	STAI (linear model)		BSQ (negative binomial model)	
	(1)	(2)	(3)	(4)
Intercept	43.69* (1.46)	45.29* (1.70)	3.28* (0.05)	3.34* (0.05)
Condition: other	−2.41 (2.22)	−3.29 (2.15)	−0.11 (0.07)	−0.13* (0.06)
Sex: male	–	−2.88 (2.07)	–	−0.14* (0.06)
B-FNE	–	0.39* (0.11)	–	0.009* (0.004)
Goodness of model fit	Adj. R^2^ = 0.002	Adj. R^2^ = 0.137	AIC = 735,	AIC = 722,
	*F*_1, 103_ = 1.17	*F*_3, 101_ = 6.52	$X_{\mathrm{df}=103}^{2}=101$	$X_{\mathrm{df}=103}^{2}=99$
*p*	0.28	<0.001	0.55^a^	0.52^a^

*Residual errors were normally distributed in (2)-(4). B-FNE was centered for interpretability*.

*^a^Test of residual deviance indicates a good fit if the *p* value is not significant (log-linear and Poisson models did not fit the BSQ data sufficiently well, but the negative binomial model was a good fit)*.

*Significant coefficient with *p* < 0.05.

To address RQ_1_, we compared levels of self, social, spatial, and overall presence between conditions using *t*-tests or the non-parametric Mann–Whitney test, depending on the distribution of residual errors (Table 3). Self-presence scores were significantly higher with the own face than with a dissimilar face, *W* = 1011, *p* = 0.023, *d* = 0.44. Other types of presence and overall presence were not significantly different (see Table 3).

## General Discussion

In the pilot study, we explored the idea that socially anxious individuals would prefer to become someone else in a social situation. Social anxiety correlated significantly with a preference for embodying a dissimilar avatar. In Study 1, we compared levels of anxiety in three experimental conditions: participants were assigned the real face, a dissimilar face, or given a face of their choice. While this study yielded no statistically significant differences in levels of anxiety, it suggested that participants embodying an assigned self avatar tended to exhibit higher levels of anxiety, followed by participants in the *choice* condition. Participants who were assigned a dissimilar avatar tended to experience the least anxiety of the three groups. Also, we identified significant differences in the sense of presence between the *self* and the *choice* conditions. Participants in the *self* condition experienced a greater sense of presence. Finally, in Study 2, we partially replicated Study 1 focusing on the *self* and *other* avatar conditions. We found significant differences in anxiety in the same direction as in Study 1: participants who were assigned a self avatar experienced 14% higher levels of anxiety measured by BSQ than participants assigned a dissimilar avatar when accounting for differences in sex and B-FNE. Yet, anxiety levels measured by STAI remained unchanged. Regarding presence, participants in the *self* condition experienced greater self-presence than those in the *other* condition.

We believe that embodying a dissimilar avatar helped participants reduce their anxiety to some extent. While the pilot study provided strong support for our hypothesis, the results of two experimental studies were more mixed. Thus, follow-up studies with a different procedure, design, or technique need to further investigate whether embodying a different self can in fact reduce anxiety. A possible explanation is that, in general, participants experienced low self-presence both in Study 1 and 2. Thus, it is possible that the process of embodiment and identification with the avatar was not strong enough to make the differences between conditions significant. In connection to this, several limitations can be pointed out. Principally, the avatars were fairly limited in terms of range of movements and face modeling. It would be preferable to render more joints such as elbows or leg movements to provide a more natural body movement to the avatar and make the reflection in the mirror appear more natural. Moreover, we used a generic male or female body for all participants, which sometimes was very different from the participant’s real body. Body shape should be taken into account in future experiments. Finally, synchronization with the movement of the avatar in the mirror was done before the virtual audience entered the virtual room. Due to technical failure of the orientation-tracking device, we did not have reliable recordings of the percentage of time participants looked at their mirror image. Future studies should use gaze behavior as a proxy for attention to the mirror image, and examine its mediating role on self-presence.

There are other limitations in the current work. In the pilot study, the avatar similarity measure that we developed should be expanded into a more complete scale. In addition, the manipulation check for facial similarity included in our questionnaire pointed at some issues with the manipulation of avatar similarity. While facial similarity was significantly higher in the *self* than *other* condition in both studies, some participants’ ratings were in the opposite direction and inconsistent with open-ended comments provided at the end of the study. The specific question wording may have confused some participants. We therefore decided not to exclude participants based on the manipulation check. Similar issues with explicit ratings were encountered in prior work on doppelgangers (Fox and Bailenson, 2009) and highlight discrepancies between survey measures of perceived similarity and actual avatar similarity. Future research should explore a better measure for manipulation check. Also, we considered trait social anxiety in all our analyses, but we used different strategies across our studies to select our participants regarding prescreening them or not for social anxiety. Other studies should examine this further and perhaps repeat similar experiments with patients diagnosed with social phobia. Study 1 presented other limitations that were fixed in Study 2 as described above.

Our findings have important theoretical and practical implications and future studies are encouraged to continue the line of research presented here. For theories of social anxiety and self-representation, the results of our study help to understand better the mechanisms underlying social anxiety. Also, more research should investigate whether alterations of self-representation should be considered as a potential positive contribution to VR exposure therapy for the treatment of social phobia. For instance, further research could examine the effectiveness of progressively increasing patient’s avatar resemblance to the real self along sessions in VRET. Therapists can leverage the findings to include the virtual self as part of the treatment. Most therapy for overcoming anxieties in VR is focused on exposure. Here, we provide a different approach based on the assumption that a negatively distorted self is at the core of social anxiety. Following this approach, we developed a technique to treat social phobia using VR based on modification of self-appearance. With this tool, therapists can help patients understand their phobia from a different perspective and work on correcting their self-image and improving their confidence in social situations.

## Conflict of Interest Statement

The authors declare that the research was conducted in the absence of any commercial or financial relationships that could be construed as a potential conflict of interest.

## Supplementary Material

The Supplementary Material for this article can be found online at http://www.frontiersin.org/Journal/10.3389/fnhum.2014.00944/abstract
