# Supplementary material for: The Relationship between Virtual Self Similarity and Social Anxiety
Source: Front Hum Neurosci. 2014 Nov 19;8:944. doi: 10.3389/fnhum.2014.00944 (PMC4237051; doi:10.3389/fnhum.2014.00944)
Supplement: Supplementary file 1 [file Data_Sheet_1.DOCX]

**Table S1**

*Correlations among trait anxiety, state anxiety, and presence measures (*N*=82)*

|  | B-FNE | Anxiety before | Anxiety during | BSQ | Self Presence | Social Presence | Spatial Presence | Overall Presence |
| --- | --- | --- | --- | --- | --- | --- | --- | --- |
| PRCA-24 | .44** | .24* | .34** | .26* | .06 | .13 | .03 | .05 |
| B-FNE |  | .36** | .37** | .40** | .05 | .01 | .09 | .06 |
| Anxiety before |  |  | .61** | .52** | .16 | .10 | .18 | .18 |
| Anxiety during |  |  |  | .66** | .05 | .02 | .01 | .03 |
| BSQ |  |  |  |  | .08 | .15 | .02 | .11 |
| Self Presence |  |  |  |  |  | .46** | .36** | .74** |
| Social Presence |  |  |  |  |  |  | .69** | .88** |
| Spatial Presence |  |  |  |  |  |  |  | .82** |
| * *p* < .05 ** *p* < .01 (2-tailed) | | | | | | | | |

**Table S2**

*Correlations among trait anxiety, state anxiety, and presence measures (*N*=105)*

|  | B-FNE | STAI | BSQ | Self Presence | Social Presence | Spatial Presence | Overall Presence |
| --- | --- | --- | --- | --- | --- | --- | --- |
| PRCA-24 | .44** | .41** | .24* | .21* | .19* | .24* | .25* |
| B-FNE |  | .36* | .28** | .06 | .13 | .20* | .16 |
| STAI |  |  | .58** | .10 | .12 | .03 | .10 |
| BSQ |  |  |  | .18 | .13 | .08 | .15 |
| Self Presence |  |  |  |  | .53** | .59** | .82** |
| Social Presence |  |  |  |  |  | .68** | .86** |
| Spatial Presence |  |  |  |  |  |  | .88** |
| * *p* < .05 ** *p* < .01 (2-tailed) | | | | | | | |
